# Peptide deformylase inhibitor actinonin reduces celastrol’s HSP70 induction while synergizing proliferation inhibition in tumor cells

**DOI:** 10.1186/1471-2407-14-146

**Published:** 2014-03-04

**Authors:** Bin Peng, Xue Zhang, Fanfan Cao, Ying Wang, Limin Xu, Lu Cao, Chunxin Yang, Maoquan Li, Georges Uzan, Denghai Zhang

**Affiliations:** 1 Sino-French Cooperative Central Lab, Shanghai Gongli Hospital, 207 Ju Ye Road, Pudong New District, Shanghai 200135, China; 2 College of Clinical Laboratory Diagnostics, Ningxia Medical University, Yinchuan 750004, China; 3 Pharmaceutical Department, Zhong Shan Hospital, Shanghai Fudan University, 136 Yi Xue Yuan Road, Shanghai 200032, China; 4 Shanghai Tenth People’s Hospital, Tongji University, 307 Yan Chang Middle Road, Shanghai 200072, China; 5 U972, Inserm, Bâtiment Lavoisier, Hôpital Paul Brousse, 12 avenue Paul Vaillant Couturier, 94807 Villejuif, Cedex France

## Abstract

**Background:**

Celastrol is a promising anti-tumor agent, yet it also elevates heat shock proteins (HSPs), especially HSP70, this effect believed to reduce its anti-tumor effects. Concurrent use of siRNA to increase celastrol’s anti-tumor effects through HSP70 interference has been reported, but because siRNA technology is difficult to clinically apply, an alternative way to curb unwanted HSP70 elevation caused by celastrol treatment is worth exploring.

**Methods:**

In this work, we explore three alternative strategies to control HSP70 elevation: (1) Searching for cancer cell types that show no HSP70 elevation in the presence of celastrol (thus recommending themselves as suitable targets); (2) Modifying HSP70-inducing chemical groups, i.e.: the carboxyl group in celastrol; and (3) Using signaling molecule inhibitors to specifically block HSP70 elevation while protecting and/or enhancing anti-tumor effects.

**Results:**

The first strategy was unsuccessful since celastrol treatment increased HSP70 in all 7 of the cancer cell types tested, this result related to HSF1 activation. The ubiquity of HSF1 expression in different cancer cells might explain why celastrol has no cell-type limitation for HSP70 induction. The second strategy revealed that modification of celastrol’s carboxyl group abolished its ability to elevate HSP70, but also abolished celastrol’s tumor inhibition effects. In the third strategy, 11 inhibitors for 10 signaling proteins reportedly related to celastrol action were tested, and five of these could reduce celastrol-caused HSP70 elevation. Among these, the peptide deformylase (PDF) inhibitor, actinonin, could synergize celastrol’s proliferation inhibition.

**Conclusions:**

Concurrent use of the chemical agent actinonin could reduce celastrol’s HSP70 elevation and also enhance proliferation inhibition by celastrol. This combination presents a novel alternative to siRNA technology and is worth further investigation for its potentially effective anti-tumor action.

## Background

Celastrol is a triterpenoid compound first identified in the plant Tripterygium wilfordii Hook F (TWHF). This herb has been used in China for many years to treat rheumatic diseases. Celastrol is an active component with many actions, among which are anti-tumor effects. It has been confirmed that celastrol can exert anti-tumor effects both *in vitro* and *in vivo* towards a variety of tumor cells with different tissue origins [1-3]. Celastrol’s anti-tumor effects are related to this agent’s ability to arrest the cell cycle and induce apoptosis [2-5].

In addition to its anti-tumor effects, celastrol also has the capacity to trigger heat shock response (HSR), causing the elevation of multiple kinds of heat shock proteins (HSPs), especially HSP70, regarded as a hallmark of HSR. Westerheide et al. demonstrated for the first time that celastrol could induce HSPs in several cell lines and suggested that it might be useful in treating neuron degenerative diseases [6]. Following this research, several groups confirmed that celastrol could indeed improve neuron degenerative alterations [7-9]. For example, in the G93A SOD1 transgenic mouse model of ALS, celastrol significantly improved motor performance and delayed the onset of ALS, in part by increasing HSP70 expression in the lumbar spinal cord neurons of celastrol-treated G93A mice [7]. The mechanism for celastrol’s HSR induction is suggested to be due to celastrol’s ability to inhibit HSP90, in turn causing HSF1 release and activation.

Though celastrol’s HSR induction can be applied to neuron degenerative disease management, for anti-tumor applications, HSR induction is an unwanted response, since the HSP elevation, especially HSP70 and HSP90, aid tumor cell survival. Reducing HSR in celastrol-treated tumor cells might enhance this agent’s anti-tumor effects. This notion is supported by the findings of Matokanovic et al., who recently proved that siRNA silencing of HSP70, a prominent molecule in celastrol-caused HSR, enhances celastrol-induced cancer cell death [10]. However, siRNA technology requires transfection, and presently is difficult to employ in clinical applications. As such, we consider that an alternative method for controlling unwanted HSR caused by celastrol is worth exploration in regards to tumor treatment.

Theoretically, there are at least three strategies to control unwanted HSR while preserving celastrol’s anti-tumor effects. The first potential method is to find cancer cell types that do not undergo HSR in celastrol’s presence, and then treat these kinds of tumors as most suitable for celastrol application. As an example, it has been suggested that some cell-type tumors, such as MCF-7 (originating from breast cancer), have no HSR when treated with celastrol [11]. A second potential method is to modify celastrol’s chemical structure to abolish HSR while maintaining anti-cancer ability. To support this idea, some researchers have suggested that the quinone methide moiety is critical to celastrol’s cytotoxic and apoptotic activity, while the acidic carboxylate group is important to heat shock response and cytoprotective activity [6]. This means that modification of celastrol’s carboxyl group might help us achieve our goal. The third potential method is to modify cells to control HSR signaling. For this strategy, we used the knowledge that siRNA can down-regulate HSP70. Since siRNA application presents clinical difficulties, we thought that inhibitors targeting the signaling proteins might block the HSR pathway and achieve the same goal. These potential targets, however, are still under investigation.

In this paper, we explore the above strategies in the following ways; first, we observed celastrol’s effects on HSR induction in tumors of different cell types. Second, we evaluated the effects of modifying celastrol’s carboxyl group on HSR induction and proliferation inhibition. Third, we observed the effects of a panel of signaling molecule inhibitors on these two celastrol actions. The results showed that the peptide deformylase inhibitor, actinonin, could reduce HSR while enhancing proliferation inhibition.

## Methods

### Materials

RPMI 1640 medium, Dulbecco’s modified Eagle’s medium (DMEM), fetal bovine serum (FBS), and streptomycin/penicillin for cell culture were obtained from PAA Laboratories (Linz, Austria). Wang resin was obtained from Synthesis Technologies Inc. (Tianjin, China). DMAP, DCC and DMF were purchased from Dikma Technologies Inc. (Beijing, China). Fmoc-L-Gly-OH and HBTU came from Tianmapharma Co., Ltd (Suzhou, China). Piperidine and NMM were obtained from Sinopharm Chemical Reagent Co., Ltd (Shanghai, China). Carboxyfluorescein diacetate, succinimidyle ester (CFSE) was from Molecular Probe (Eugene, OR) and 7-Amino-actinomycin D (7-AAD) was purchased from Anaspec (San Jose, CA). Novobiocin (NB) and dimethyl sulfoxide (DMSO) were purchased from Sigma (St. Louis, MO). 17-allylamino-17- demethoxygeldanamycin (17-AAG) was obtained from Invivogen (San Diego, CA). Anti-HSF1 antibody (Cat. 4356) was obtained from Cell Signaling Technology Inc. (Boston, MA). Anti-HSP70, anti-HSP40, anti-HSP90, and anti-p-HSF1 (Ser326) antibodies were purchased from ENZO Life Sciences Inc. (Farmingdale, NY, USA). Anti-p-HSF1 (Ser303) was purchased from Abcam (Cambridge, MA, US). Nuclear and Cytoplasmic Protein Extraction Kit, BCA protein assay reagent kit and Beyo ECL Plus for western blot were purchased from Beyotime Biotechnology (Jiangsu, China). Phosphatase inhibitor cocktail tablets were obtained from Roche (Mannheim, Germany). All reagents were stored according to manufacturer recommendations.

Celastrol was extracted as previously reported by us [12]. Celastrol and 17-AAG were dissolved in 50 mM and 1 mg/ml in DMSO, respectively. NB was dissolved in ddH_2_O. All of these drugs were stored at -20°C and used within 3 months of preparation. The stored solution was further diluted with RPMI 1640 medium or DMEM to a proper lower concentration immediately before experiments.

### Cell culture and treatment

The seven kinds of human cancer cell lines used in this study were obtained from the Shanghai Cell Bank of the Chinese Academy of Sciences, including breast cancer cell lines MCF-7 and MDA-MB-468, prostate cancer cell line PC3, hepatic cancer cell line HepG2, leukemic cell lines THP-1, U937, and NB4. Cells were maintained in RPMI 1640 or DMEM supplemented with 10% FBS, 100 IU/ml penicillin and 100 μg/ml streptomycin in a humidified 5% CO_2_ incubator at 37°C. Exponentially growing cells were used for experiments. Cells were seeded into 96-well or 6-well culture plates followed by exposure to the indicated doses of celastrol, 17-AAG, or NB for the indicated times. The culture medium with DMSO (vehicle) served as control. The final concentration of DMSO never exceeded 0.1%. Each experiment was repeated at least three times.

### Western blot

Cells were incubated in lysis buffer and cleared by centrifugation at 13,000 × g for 10 min. For the phosphorylation protein assay, phosphatase inhibitor was added to suppress the activity of phosphatase. The extraction of cytoplasmic and nuclear protein was performed according to product manufacturer instructions. A BCA protein assay reagent kit determined protein concentrations. Aliquots of samples were subjected to 10% SDS-polyacrylamide gels and then transferred to polyvinylidenedifluoride (PVDF) membranes. Membranes were probed with the indicated antibodies. Detection was accomplished using corresponding horseradish peroxidase (HRP)-conjugated secondary antibodies followed by development with Beyo ECL Plus; pictures were captured by G: BOX iChemi XR (Syngene Inc., UK).

### Immunofluorescence

Cellular localization of HSF1 was assessed by immunofluorescence. PC3 monolayers grown on coverslips were exposed to 600 nM of celastrol for 10 min. At the end of the experimental period, PC3 monolayers were washed twice in cold PBS and fixed with 2% paraformaldehyde for 20 minutes. After being permeabilized with 0.1% Triton X-100 in PBS at room temperature for 20 minutes, monolayers were then incubated in blocking solution composed of bovine serum albumin and normal donkey serum in PBS for 1 hour. Cells were then labeled with primary antibodies in blocking solution overnight at 4°C. After being washed with PBS, the cells were incubated in Alxa Fluor 488 conjugated-secondary antibody for 1 hour at room temperature. Before being mounted on microscope slides, cells were incubated in PI at 37°C. Immunolocalization of HSF1 protein was visualized using an Olympus fluorescence microscope (Tokyo, Japan).

### Synthesizing structural analogues for celastrol

The celastrol analogue we used was celastrol attached to tri-petite of cylcine, a structure with similar length but less complicated than those used by Klaić et al to modify celastrol [13]. It was synthesized as follows: Wang resin was put into a tube and DMF (15 mg/ml) was added. After shaking for 30 min, the solution was filtered out. Then, Fmoc-L-Gly-OH, DMAP, DCC, and DMF were added sequentially, after reaction with shaking for 30 min, acetic anhydride was added as a blocker. 20% piperidine in DMF was used to remove the protective group Fmoc, followed by successive washing with DMF, methanol, and DMF, then, Fmoc-L-Gly-OH, HBTU and NMM were added, and a condensation reaction was carried out for 30 min, and the system was washed and 20% piperidine in DMF used to remove the Fmoc group. The system was washed again, and the condensation reaction repeated until the third glycine was coupled. Then, celastrol was added and connected to the tri-peptide of glycine through another 30 min round of condensation reaction in the presence of HBTU and NMM. The reaction system was washed as in previous steps and additionally washed with DCM three times. The resin was dried, and the synthesized product was cut and purified with semi-preparative HPLC (SHIMADZU, Japan). Celastrol analogues and tri-peptide alone were identified by API 150EX Mass Spectrometer System (PE Sciex Corp. USA).

### Cell counting by flow cytometry (FCM)

At the end of the indicated time points, cells were collected and the cells were enumerated. Accurate enumeration was carried out by FCM based on a single-tube platform with self-made cell-Beads as internal controls, a method originally reported by Harrison et al. [14] and modified by us [15]. Briefly, samples were collected followed by the addition of a known number of self-made CFSE-containing Cell-Beads. Before analysis by FACScalibur flow cytometer (Becton-Dickinson, CA), 7-AAD was added with a final concentration of 1 μg/ml for separating dead cells. The FL1 detector was used for discrimination between Cell-Beads and tested cells. The FL3 detector was used to discriminate vital cells from dead. 10,000 events were detected. The number of vital (or dead) cells was calculated using the following equation:

$$\begin{array}{l} \mathrm{Number}\;\mathrm{of}\;\mathrm{vital}\left(\mathrm{dead}\right)\mathrm{cells}=[\mathrm{number}\;\mathrm{of}\;\mathrm{vital}\left(\mathrm{dead}\right)\;\mathrm{cells}\;\mathrm{detected}/\\ \;\;\;\;\;\mathrm{number}\;\mathrm{of}\;\mathrm{Cell}-\mathrm{Beads}\;\mathrm{detected})]\\ \;\;\;\;\;\times \mathrm{number}\;\mathrm{of}\;\mathrm{Cell}-\mathrm{Beads}\;\mathrm{input} \end{array}$$

### Drug interaction study

U937 cells were incubated with different doses of celastrol or the inhibitors for 24 h, and the number of vital, dead, and total cells were counted by FCM. D_m_ value (the median-effect dose or concentration) of each drug was obtained using Calcusyn 2.0 software. According to the D_m_ value, various concentrations of the single agents and the combinations with a fixed constant ratio were tested. The drug interaction study was analyzed with Calcusyn 2.0 software.

### Statistics

Data in this study are presented as mean ± SD. Student’s *t*-test or One-way analysis of variance (ANOVA) was used for statistical evaluation of significant differences among the groups using SPSS statistics 17.0 software. A value of *P* < 0.05 was considered to be statistical significance. Experiments were repeated at least three times.

## Results

### Celastrol induced HSP70 and other HSPs expression in multiple kinds of cancer cells, accompanied by phosphorylation and nuclear accumulation of HSF1

**Figure 1:**
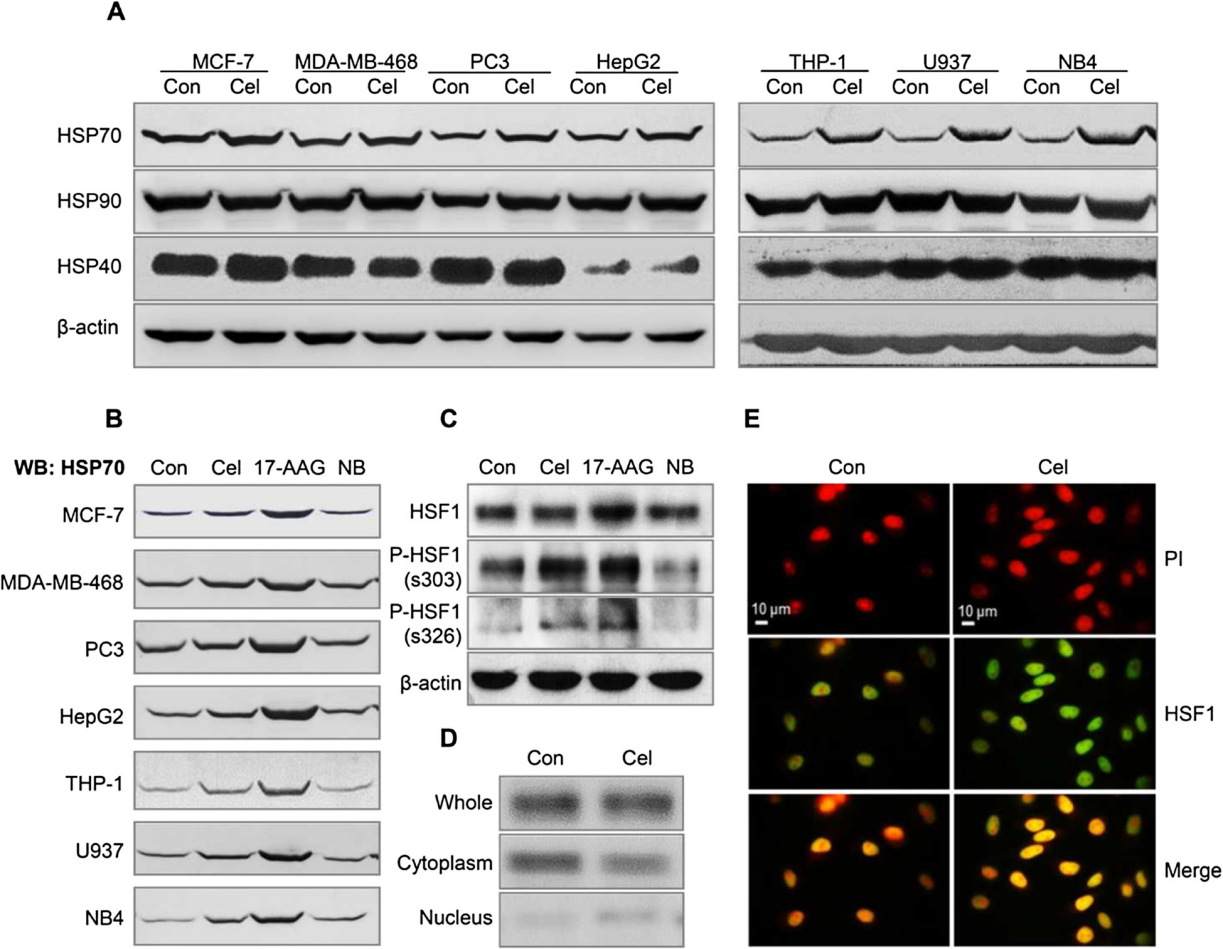
**The effects of celastrol on HSPs expression in cancer cells accompanied by HSF1 activation. (A)** Seven kinds of cancer cell lines were treated with 600 nM celastrol for 24 h. The culture medium with DMSO (vehicle) served as control for drugs. After treatment, cells were harvested and total protein was isolated. The levels of HSPs (HSP70, HSP90, and HSP40) were assayed by western blot (WB). **(B)** The cell lines were treated with 600 nM of celastrol, 17-AAG, or NB for 24 h. WB detected HSP70 expression. **(C)** Phosphorylation of HSF1 detected in PC3 cell. The cells were treated with 600 nM of celastrol, 17-AAG, or NB for 10 min. The total protein was subjected to SDS-PAGE. The activation of HSF1 was probed with anti-HSF1, and phosphorylated-HSF1 (s326 and s303) antibodies. **(D)** Distribution of HSF1 in cytoplasm and nuclei in PC3 cells. The cells were treated with 600 nM of celastrol for 10 min. The proteins in total cells, plasma, and nucleus were extracted and WB detected the levels of HSF1. **(E)** Nuclear accumulation of HSF1 in PC3 cells. The cells were grown on coverslips for at least 24 h in culture medium, then the medium was replaced by one of 600 nM of celastrol for 10 min. HSF1 was detected by immunofluorescence using an anti-HSF1 (green) antibody which mainly recognizes HSF1 in nuclei. DNA was stained with PI (red). The magnification was ×1000. Con: control; Cel: celastrol; 17-AAG: 17-allylamino-17- demethoxygeldanamycin; NB: novobiocin.

For the first strategy, we selected 7 cancer cell types and detected the expressions of HSPs in these cancer cell lines when treated with 600 nM celastrol for 24 h. (This dose was selected by pre-experimental screening, a process that was also applied to HSP90 inhibitors 17-AAG and NB). Western blot results showed that celastrol use resulted in significant up-regulation of HSP70 in all 7 of these cancer cell lines (Figure 1A). Celastrol treatment-caused HSP90 elevation was not found in 5 of the 7 tested cell lines, excepting PC3 and NB4. Another member of the HSP family, HSP40, was not significantly affected by celastrol (Figure 1A).

The HSP90 inhibitor 17-AAG has been reported to induce HSPs while another HSP90 inhibitor, NB, cannot [16,17]; thus these two agents were chosen as positive and negative agents to confirm the reliability of our experimental system. The effects of 17-AAG and NB in inducing HSP70 were observed in these 7 cell lines. The results showed that 17-AAG could significantly induce HSP70 elevation, while NB could not (Figure 1B). These results for 17-AAG and NB were consistent with previous reports on these two agents, indicating that our system is reliable for evaluating celastrol’s ability to induce HSP70.

The above results suggested that the induction of HSPs, especially HSP70, is a common action of celastrol. That celastrol’s effects are not cancer cell type dependent might be explained by another suggested effect of celastrol, HSF1 activation. HSF1 is ubiquitous molecule in mammalian cells, and its activation could cause HSP70 expression. Therefore, we observed the effects of celastrol on HSF1 in PC3 cells. Western blot analysis showed that celastrol treatment at 600 nM for 10 min could significantly phosphorylate ser303 and ser326 on HSF1 when compared to the control (Figure 1C). 17-AAG was also found to cause HSF1 phosphorylation, while NB did not. Then, we observed the effects of celastrol on HSF1 distribution. The total amounts of HSF1 were similar in both the DMSO-treated control and celastrol-treated cells, however, a reduction in cytoplasm and elevation in the nuclear extract of this protein were revealed in cells treated with celastrol at 600 nM for 10 min (Figure 1D), implying that some of the HSF1 was rapidly relocated from cytoplasm to nuclei after celastrol was loaded. Consistent with this analysis, the antibody (Cat. 4356 from Cell Signaling Technology Inc., specific to the nuclear form of HSF1) presented more intensified nuclei staining in cells treated by celastrol for 10 min, indicating celastrol’s nuclear accumulation of this transcript factor (Figure 1E). Phosphorylation and cellular distribution detections showed that celastrol rapidly activated HSF1 in our system.

### Modification of celastrol’s carboxyl group abolished its HSP70 induction effects as well its proliferation inhibition

**Figure 2:**
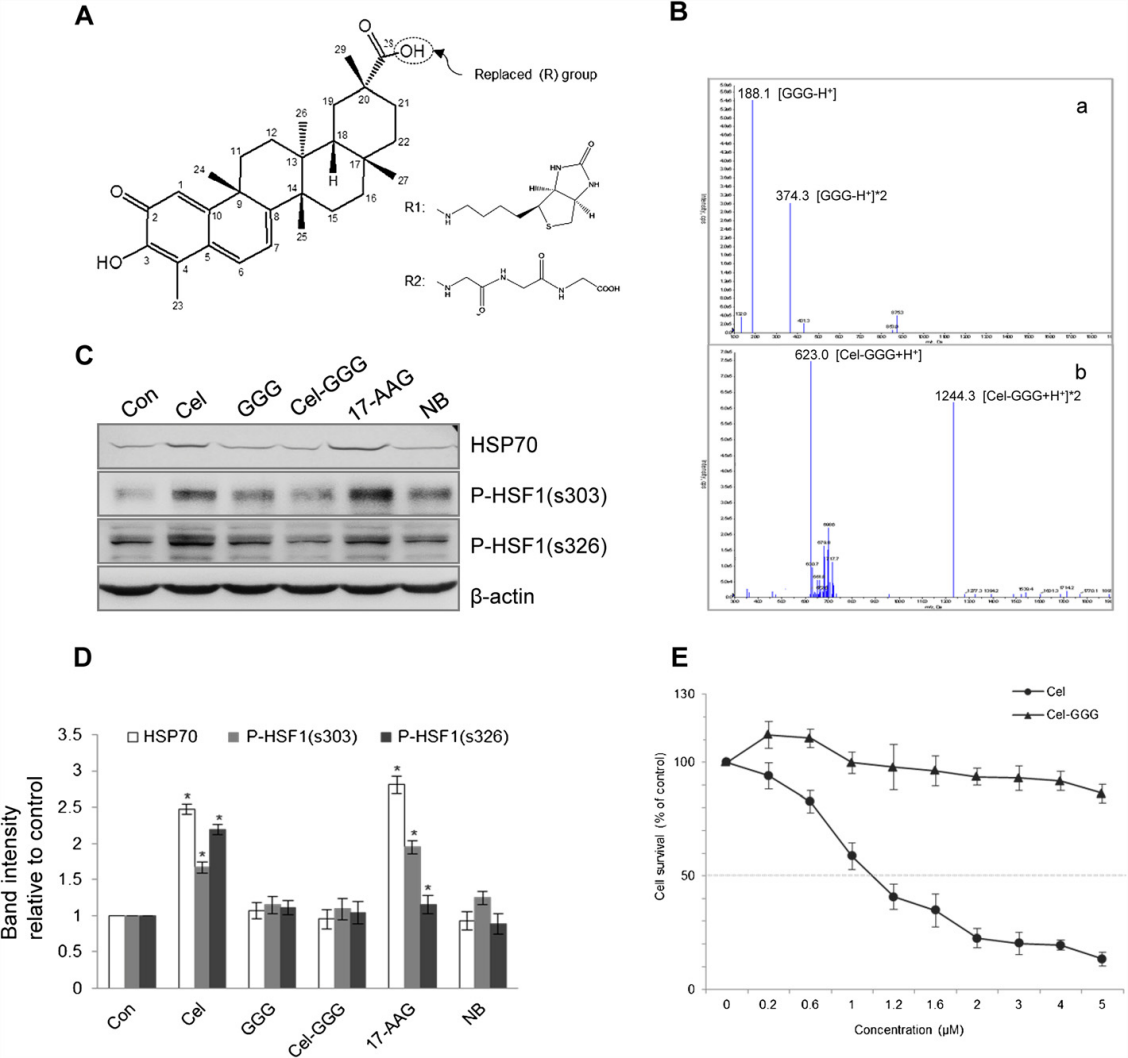
**The effects of celastrol carboxyl group modification on HSP70 induction. (A)** Structure of celastrol and its modifications. The circled point indicates the modification location. R1 represents one of the structures that used by Klaić et al. to create celastrol analogues, while R2 is tri-peptide of glycine, the analogue used in this paper. **(B)** Identifications of (a) tri-peptide of glycine and (b) the celastrol analogue were `performed by mass spectrum analysis. **(C)** Effects of the celastrol analogue on HSP70 expression and HSF1 phosphorylation. U937 cells were treated with 600 nM of celastrol, GGG, Cel-GGG, 17-AAG, or NB for 24 h. For phosphorylation assay, cells were incubated with drugs for 10 min. WB detected HSP70 and p-HSF1 (s303 and s326). **(D)** The histogram shows western blot band density statistics for part C. * represents *P* < 0.05 *vs*. control. **(E)** Effect of the celastrol analogue on proliferation inhibition. Cells were treated with 600 nM of celastrol or its analogue for 24 h, and cellular survival was analyzed by FCM according to our previous report. GGG: tri-peptide of glycine; Cel-GGG: celastrol joined with tri-peptide of glycine through peptide formation.

For the second strategy, we modified celastrol’s carboxyl group (suggested to be responsible for HSP70 induction) by attaching tri-peptide of glycine via peptide bond formation (Figure 2A), and then observed the effects of this modified celastrol on HSP70 induction and cellular survival. The purity of celastrol analogues or tri-peptide of glycine was over than 95% (Figure 2B). The results showed the modified celastrol did not induce HSP70 and phosphorylate HSF1 (Figure 2C and D), but also could not inhibit proliferation (Figure 2E). Since this attached structure, when used alone, showed no effects on cellular HSP70 levels, disability of the modified celastrol to inhibit proliferation should not be due to the attached tri-peptide of glycine (Figure 2C and D). We also tried modifying celastrol’s carboxyl group by attaching one glycine rather than tri-peptide of glycine, but the results were the same (data not shown).

### Peptide deformylase inhibitor actinonin reduced HSP70 induction while synergizing celastrol’s proliferation inhibition

**Figure 3:**
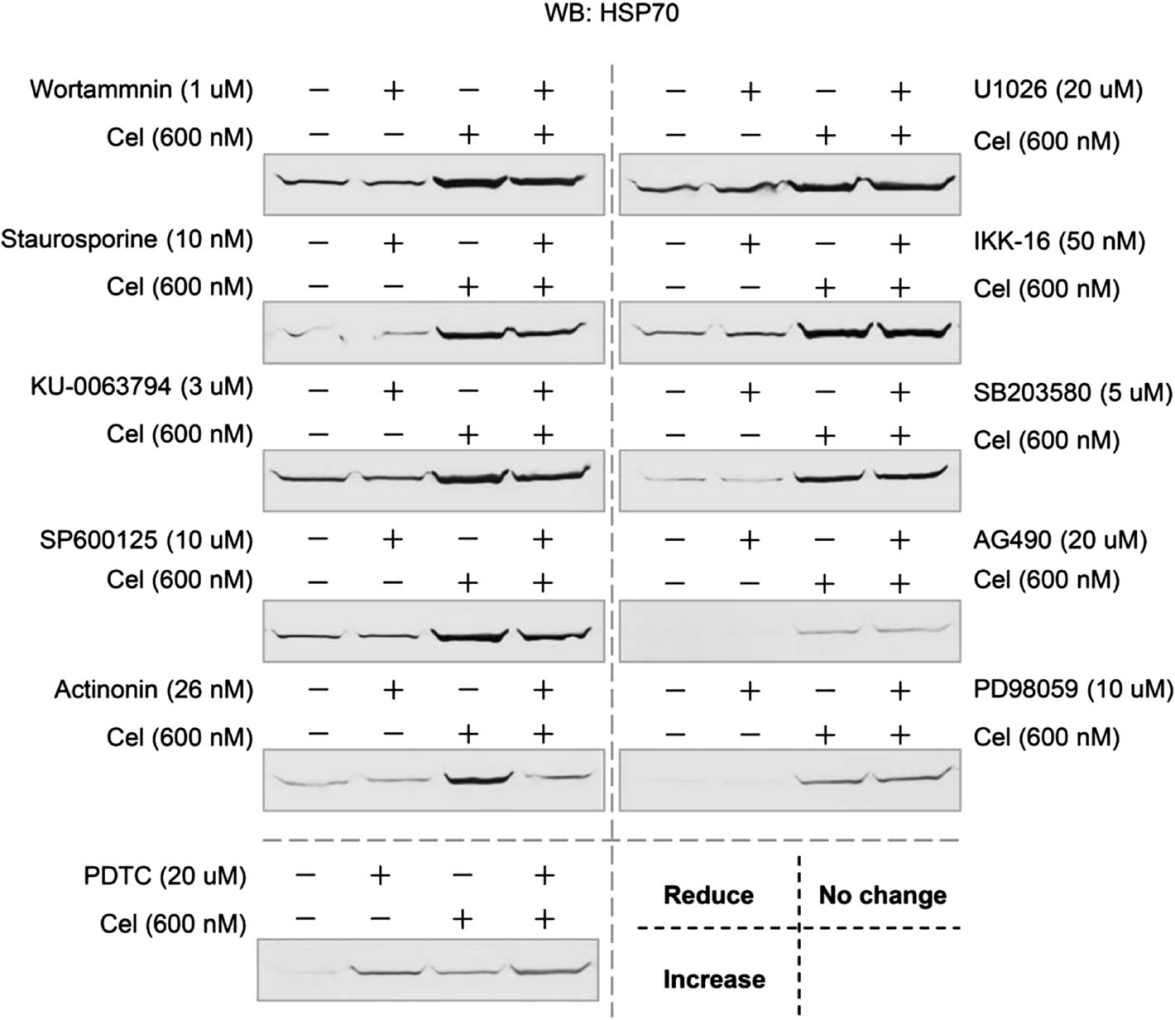
**The effect of molecular signaling inhibitors on celastrol’s HSP70 induction.** After incubation with the indicated doses of inhibitors for 1 h, U937 cells were treated with 600 nM of celastrol for 24 h. The HSP70 expressions were assayed by WB.

In the third strategy, we tried to find signaling protein inhibitors that might specifically inhibit the HSP70 induction pathway but not interfere with celastrol’s proliferation inhibition. 11 inhibitors towards 10 signaling molecules were observed, including PI3K/AKT inhibitor wortmanin, PKC inhibitor staurosporine, mTOR1/2 inhibitor KU-0063794, JNK inhibitor SP600125, peptide deformylase (PDF) inhibitor actinonin, MEK1/2 or MEK1 inhibitor U1026 and PD98059, IKK inhibitor IKK-16, p38 MAPK inhibitor SB203580, and JAK inhibitor AG490, NF-κB inhibitor PDTC. 5 of the inhibitors significantly reduced celastrol-induced HSP70: PI3K/AKT inhibitor, PKC inhibitor, mTOR1/2 inhibitor, JNK inhibitor, and PDF inhibitor. Among these, the PDF inhibitor, actinonin, had the most obvious HSP70 reduction effect. NF-κB inhibitor PDTC exerted an enhancing action on HSP70 induction. The remaining 5 inhibitors had no significant effects on celastrol-induced HSP70 (Figure 3).

**Figure 4:**
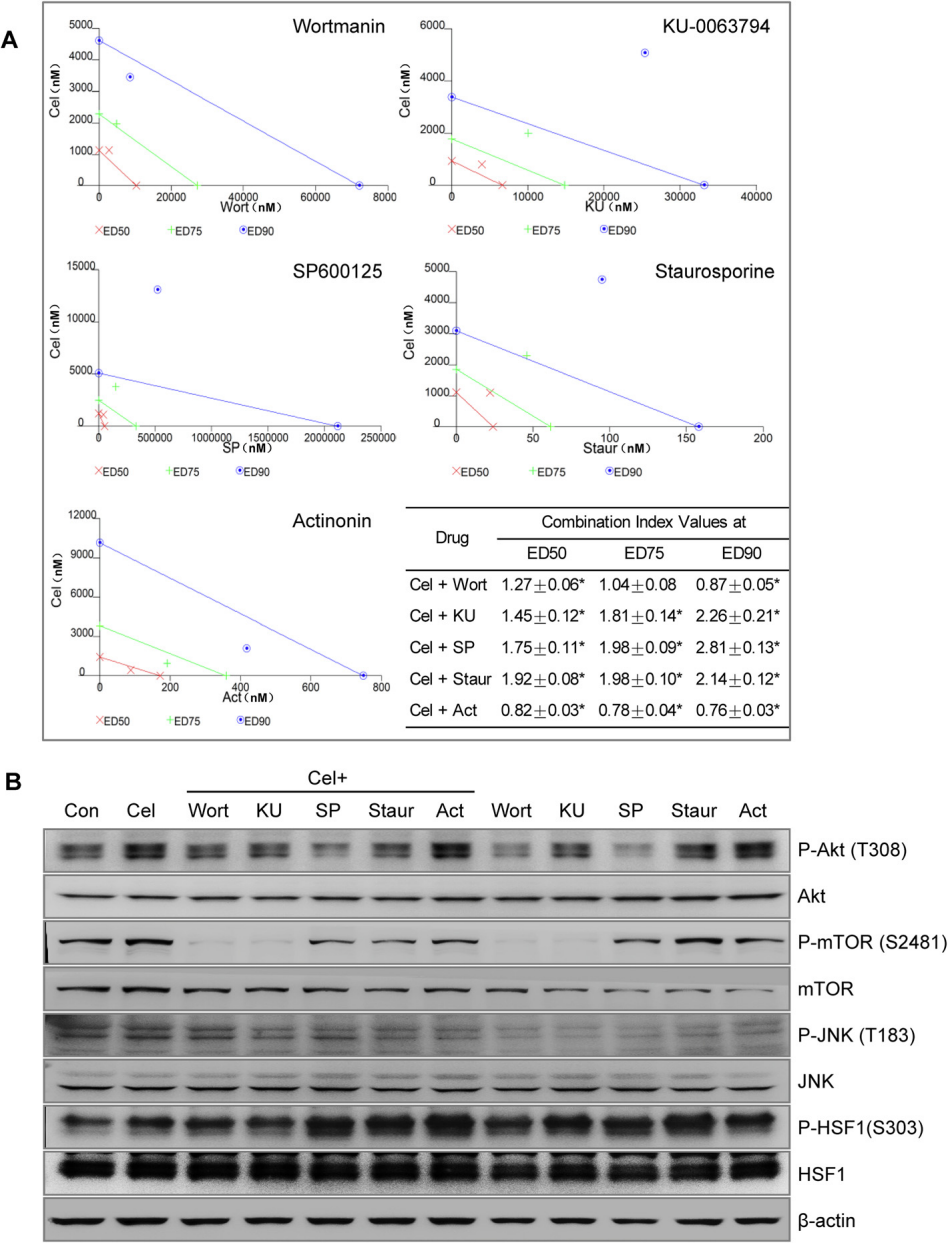
**The effects of some signaling inhibitors on proliferation inhibition and HSP70 induction-related molecules activations. (A)** Isobologram analysis for combined effects of celastrol and the inhibitors reducing celastrol’s HSP70 induction on proliferation inhibition. U937 cells were incubated with different doses of celastrol or the inhibitors for 24 h, and the numbers of vital, dead, and total cells were counted by FCM. D_m_ value (the median-effect dose or concentration) of each drug was obtained using Calcusyn 2.0 software. According to the D_m_ value, various concentrations of single agents and agent combinations with a fixed constant ratio were tested. The isobologram analysis was analyzed with Calcusyn 2.0 software. Combination index (CI) = 1 refers to additive effect, CI < 1 refers to synergism, and CI > 1 means antagonism. ED50, ED75, and ED90 represent effective doses of 50%, 75%, and 90% inhibition, respectively. The values were shown as mean ± SD. * *P* < 0.05 vs. 1. **(B)** Effects of inhibitors reducing celastrol’s HSP70 induction on the HSP70 regulation signaling pathway. U937 cells were pre-incubated with the inhibitors for 1 h, followed by treatment with 600 nM celastrol for 10 min. Total proteins were extracted by lysis buffer with phosphorylation inhibitor. WB assayed phosphorylated or total signaling molecules.

To determine if the inhibitors that reduced HSP70 expression could enhance the proliferation inhibition caused by celastrol, we observed the combinative effects of celastrol and these inhibitors in U937 cells. The results showed that only the combination of actinonin and celastrol had a synergetic action in proliferation inhibition (based on combination index (CI) values <1) (Figure 4A). The other four inhibitors (PI3K/AKT inhibitor, mTOR1/2 inhibitor, JNK inhibitor, and PKC inhibitor) reduced HSP70 levels, but also antagonized celastrol’s proliferation inhibition (CI value >1) (Figure 4A).

Next, we observed the relationship between the inhibitors for HSP70 induction and HSF1 activation. When treated with celastrol for 10 min, the phosphorylations of AKT, mTOR and HSF1 in U937 cells were dramatically increased. JNK phosphorylation was slightly elevated. The inhibitors of PI3K/AKT, mTOR, JNK, PKC could reduce AKT and mTOR phosphorylation (activations) caused by celastrol, but had no obvious or elevating effect on p-HSF1. Interestingly and strikingly, the PDF inhibitor, actinonin, significantly enhanced celastrol-induced HSF1 phosphorylation (Figure 4B). This demonstrates that these compatible HSP70 inhibitors worked downstream of HSF1 activation.

## Discussion

In this work, we tried three strategies to get rid of the unwanted HSR in celastrol’s anti-tumor application, and found that the peptide deformylase inhibitor, actinonin, reduced HSP70 while enhancing celastrol’s proliferation inhibition.

Over a dozen reports [18-20] have confirmed the Westerheide et al. assertion that celastrol could induce HSPs [6], but there is one report in which celastrol did not increase HSP70, and this was in human breast cancer cell line MCF-7 [11]. Therefore, our first strategy to avoid unwanted HSP70 induction in antitumor celastrol application was to find some specific cancer cell types showing no HSR in celastrol’s presence (cancer cells of this type would be most suitable for celastrol application). We chose 7 cancer cell lines of different tissue origin, including MCF-7, as test cells to evaluate celastrol’s ability to induce HSPs. Each of the tested cell lines showed HSP70 elevation when treated with celastrol. Since the discrepancy between our MCF-7 results and the Matts et al. report might be due to the differences in the experimental systems, we verified our system’s reliability in evaluating agent HSPs-inducing ability by carrying out contemporaneous observation of the effects of 17-AAG and NB. Because 17-AAG and NB are widely accepted as an HSP70 inducer and non-inducer, respectively, they were chosen as positive and negative controls. In our experimental system, 17-AAG showed strong induction ability, while NB did not. These results also agree with previous reports about 17-AAG and NB, and acted to verify our system’s reliability.

We also explored the molecular mechanism for celastrol-induced HSP70 expression, and found that celastrol could activate HSF1. With the ubiquitous expression of HSF1 and HSP90 in the different cells we tested, it is easy to understand HSP70 induction as a general celastrol effect. Because of this, it is hard to select specific cancer cell types without celastrol-caused HSP70 elevation, and it stands to reason that this first strategy is probably untenable.

Our second strategy was based on the suggestion that celastrol’s carboxyl group is responsible for this agent’s HSP70 induction [6]. We found that modification of the carboxyl group could indeed abolish celastrol’s HSP70-inducing effects; however, the anti-tumor effects were also abolished in modification. The modified celastrol’s inability to act on tumors might be due to the structural analogue’s inability to enter cells, but this possibility was ruled out by a simultaneous test of a liposome agent with modified celastrol. Hence, our second strategy to control HSP90 induction through structural modification was also fruitless. We and others have reported that HSP70 induction and proliferation inhibition were both related to celastrol’s HSP90 inhibition [10,21]. Moreover, we recently used molecular docking to find the role of celastrol’s carboxyl group in HSP90 binding, the result indicating a novel binding pocket in HSP90 dimers for celastrol in which the carboxyl group formed two salt bonds with HSP90’s residues (these results will be published in another paper). This result highlighted the importance of the carboxyl group and gave explanation to our unsuccessful modification results.

With the first two strategies unsuccessful, we tried a third method in which we found some inhibitors that could specifically tune down celastrol’s HSP70-inducing arm while not affecting or possibly enhancing the proliferation-inhibition arm. Most of the inhibitors we used have been reported as celastrol-activated [22-27]. We found the inhibitors toward PI3K, AKT, mTOR, and JNK could effectively reduce celastrol-caused HSP70 induction, however, these inhibitors also caused reductions in celastrol’s proliferation inhibition ability.

The PDF inhibitor, actinonin, not only reduced HSP70 expression, but also synergized celastrol’s proliferation inhibition. Actinonin is a streptomyces-derived antibiotic, and in addition to inhibiting peptide deformylase, it was also recently shown to interact with and inhibit aminopeptidase N (APN)/CD13 [28], meprin α [29], and MMP-2 [28]. It has been clinically tested as a new anti-bacterial drug, and its antitumor effects have also attracted research attention [30]. Actinonin’s mechanism for affecting celastrol’s action is not explained in this study.

We also found that the 5 inhibitors that could inhibit celastrol-induced HSP70 elevation did not inhibit HSF1 phosphorylation, indicating that these inhibitors worked downstream of HSF1 activation. The exact mechanism for blocking HSP70 induction remains for further investigation.

Nevertheless, our results discovered a novel and practical alternative to siRNA technology in reducing celastrol-caused HSP70 elevation and enhancing celastrol’s anti-tumor effects. Since actinonin itself is an anti-tumor agent [30], its combinative use with celastrol in anti-tumor applications is suggested here.

## Conclusions

In this work, we found that HSP70 induction might be a general response of different cancer cells to celastrol treatment, and thus it may be impractical if not impossible to base celastrol application on a pick-and-choose strategy. We also found that modification of celastrol’s carboxyl group can control this agent’s HSP70 induction action, yet celastrol’s anti-tumor effects were also prevented. Lastly, we found co-use of celastrol and actinonin could reduce unwanted HSP70 induction and enhance celastrol’s tumor proliferation inhibition, and thus propose this novel method as a way to enhance celastrol’s anti-tumor effects.

## Competing interests

The authors declare no competing interests.

## Authors’ contributions

BP carried out the immunoassays, participated in the design of the study, performed the statistical analysis and drafted the manuscript. XZ participated in western blot and immunofluorescence. FC carried out cell counts. YW carried out western blot. LX participated in cell culture. LC helped to collect data. CY participated in drug structure modification. ML participated in design of the study. GU participated in study design and helped to draft the manuscript. DZ conceived of the study, participated in its design and coordination, and helped to draft the manuscript. All authors have read and approved the final manuscript.

## Pre-publication history

The pre-publication history for this paper can be accessed here:

http://www.biomedcentral.com/1471-2407/14/146/prepub
